# Effect of Human Activity and Presence on the Behavior of Long-Tailed Macaques (*Macaca fascicularis*) in an Urban Tourism Site in Kuala Selangor, Malaysia

**DOI:** 10.3390/ani14081173

**Published:** 2024-04-13

**Authors:** Mahbod Entezami, Fiqri Mustaqqim, Elizabeth Morris, Erin Swee Hua Lim, Joaquín M. Prada, Sharmini Julita Paramasivam

**Affiliations:** 1 School of Veterinary Medicine, Faculty of Health and Medical Sciences, University of Surrey, Daphne Jackson Road, Guildford GU2 7AL, UK; me00286@surrey.ac.uk (M.E.); lizziemorris9501@gmail.com (E.M.); j.prada@surrey.ac.uk (J.M.P.); 2 School of Postgraduate Studies, Perdana University, Serdang 43400, Malaysia; 3 Abu Dhabi Women’s College, Higher Colleges of Technology, Abu Dhabi 41012, United Arab Emirates; erinlimsh@gmail.com; 4 Centre for Research Excellence, Perdana University, Serdang 43400, Malaysia; 5 Animal Neighbours Project, School of Veterinary Medicine, University of Surrey, Daphne Jackson Road, Guildford GU2 7AL, UK

**Keywords:** human primate interaction, behavior, observation, urban tourism, urban wildlife, macaque, activity budget

## Abstract

**Simple Summary:**

Monkeys in urban spaces are often labeled as ‘pests’ by people who share spaces with them, mainly driven by their behavior to adapt and survive in a human-dominated environment. In Malaysia, there has been an increase in complaints about urban monkeys, which drives management strategies mainly to reduce human populations that impact the animals’ welfare and conservation. Understanding the dynamics between monkeys, people, and the urban ecosystem is the first step to identifying the drivers of the complaints. This study investigates the types of ecological activities of the long-tailed macaque (*Macaca fascicularis*) at an urban tourism site and how human activity influences it. Monkeys were impacted negatively by the presence of humans. Less affiliative interactions were performed when human traffic was high; for example, less social behavior was seen in the group. The monkeys also used anthropogenic structures predominantly when people were present and would spend time on natural structures when people were not. This study supports evidence that monkeys alter behaviors to adapt to living in urban spaces. A structured management plan needs to consider these dynamics to manage complaints.

**Abstract:**

The increasing overlap of resources between human and long-tailed macaque (*Macaca fascicularis*) (LTM) populations have escalated human–primate conflict. In Malaysia, LTMs are labeled as a ‘pest’ species due to the macaques’ opportunistic nature. This study investigates the activity budget of LTMs in an urban tourism site and how human activities influence it. Observational data were collected from LTMs daily for a period of four months. The observed behaviors were compared across differing levels of human interaction, between different times of day, and between high, medium, and low human traffic zones. LTMs exhibited varying ecological behavior patterns when observed across zones of differing human traffic, e.g., higher inactivity when human presence is high. More concerning is the impact on these animals’ welfare and group dynamics as the increase in interactions with humans takes place; we noted increased inactivity and reduced intra-group interaction. This study highlights the connection that LTMs make between human activity and sources of anthropogenic food. Only through understanding LTM interaction can the cause for human–primate conflict be better understood, and thus, more sustainable mitigation strategies can be generated.

## 1. Introduction 

Urbanization significantly alters land use, leading to habitat fragmentation and increased human–wildlife encounters [1,2,3]. The Asia-Pacific region is one of the most rapidly developing regions in the world, with 90% of future urbanization predicted to occur in the region [4]. Furthermore, this region contains almost half the globe’s biodiversity hotspots [5], so urbanization is commonly associated with habitat fragmentation. Due to growing anthropogenic demands, non-human primate (hereafter referred to as primates) numbers are declining [6], and their habitats have undergone extensive loss. Primates adapt their daily behaviors in response to environmental conditions to ensure their survival. Their activity budgets are influenced by various environmental factors, such as diet, distribution, and availability of food sources [7,8]. 

In Southeast Asia, long-tailed macaques (LTMs) (*Macaca fascicularis*) are known as ‘opportunistic’ and ‘adaptive’ but are highly affected by human activities. This has contributed to the declining trend of the population, listed as ‘Endangered’ by the IUCN [9,10,11]. Because of the distribution of LTM across regions, their behavior, social, organization, habitat usage, morphology, and genetics are varied [12,13]. LTM range covers areas throughout Southeast Asia and Peninsula Malaysia, predominantly in tourist sites such as Kuala Selangor [14]. 

LTMs spend the majority of their time traveling/moving, foraging, resting, and affiliative interacting, e.g., grooming [15,16]. As omnivores, their diet is vast, and feeding behavior takes up a large portion of the daily activity budget [17,18]. This flexibility in behavior is a means for primates to survive in changing environments. Living close to humans means anthropogenic food sources are attractive options to primates, as their situational density allows for energy conservation when obtaining food. The reported list of ‘pest primates’ has increased with the increasing conversion of natural habitats into agricultural land [19]. Of these ‘pest primates’, macaques could be considered most successful at exploiting the human–primate interface due to their vast geographical distribution and taxonomic diversity [20]. Traditionally, a commensal relationship between macaques and humans has existed across Asia, facilitated by cultural and religious beliefs that worship primates [10,21], allowing for tolerance and protection of macaques in these areas [20]. However, the increasing overlap of resources jeopardizes this traditional relationship [22,23]. 

Studies have investigated the changes in the behavior of primates in altered habitats to understand how they have adapted and the impact on their well-being [14,24,25,26,27]. It is also important to consider the effect of environmental pressures, e.g., tourism demand, that primates face when enacting sustainable conservation practices. 

Ecotourism plays a significant role in generating tourism income in many tropical regions [28]. This trend has particular implications for the well-being of primates, which are commonly at the center of such ecotourism efforts. As ecotourism gains traction in countries with primate habitats, the potential negative impacts of frequent human–primate interactions have raised concerns among researchers [29,30,31,32]. Habituation of primates to human presence, a prerequisite for enabling tourists to observe them closely [33], may inadvertently lead to changes in their ecological behavior. Primates have been observed to modify their foraging habits due to recurrent interactions with tourists [34]. Furthermore, a rise in both intragroup and human-directed aggression has been documented [35], posing risks of injury to both humans and primates. 

In Malaysia, there is an annual increase in the number of reports of human–wildlife conflict [36], with LTM receiving the highest complaints by the Department of Wildlife and National Parks Peninsular Malaysia (DWNP), followed by wild boar (*Sus scrofa*), common palm civet (*Paradoxurus hermaphroditus*), and elephants (*Elephas maximus*) [37]. Malaysia’s total land mass is covered by approximately 45% forest, and of this, only 20% is undisturbed and designated for wildlife protection [38], putting increasing pressure on wildlife populations. While the adaptation of macaque species to urbanization has benefitted their survival, considerable variation exists across individuals around their willingness to co-exist with people and exploit anthropogenic environments [20]. Current management strategies for dealing with public complaints about LTM include managing population sizes through translocation and culling to reduce interaction [37]. Strategies such as these play a part in the reduced population of LTMs in the wild. While effective from a complaint reduction perspective, it does nothing to promote long-term coexistence that ensures the conservation and welfare of the species. 

This study investigates the effects that human presence has on the activity budget of LTMs in Kuala Selangor, Malaysia, by analyzing scan sampling data. This study aimed to recognize factors that affect LTM behavior in an anthropogenic area. It was hypothesized that (a) high human presence will negatively affect the behavior of LTMs, (b) LTMs utilize anthropogenic structures more when people are present, and (c) LTM’s activity budget varies according to the level of anthropogenic exposure (low, medium, and high traffic zones). 

## 2. Materials and Methods 

### 2.1. Study Site and Study Subjects 

Data collection was carried out in Bukit Melawati, Kuala Selangor, an urban tourism site known not only for its cultural history and scenery but also for its unique ability for tourists to encounter habituated free-ranging primates, the Selangor silvered langur (*Trachypithecus selangorensis*) and LTMs. The study subjects were LTM, and social groups were not identified in this study as they were large, very dispersed, and overlapped in home ranges while living in sympatry with the langurs. All aged individuals were observed except nursing infants. Additionally, age–sex categories were not considered in this study. 

**Figure 1:**
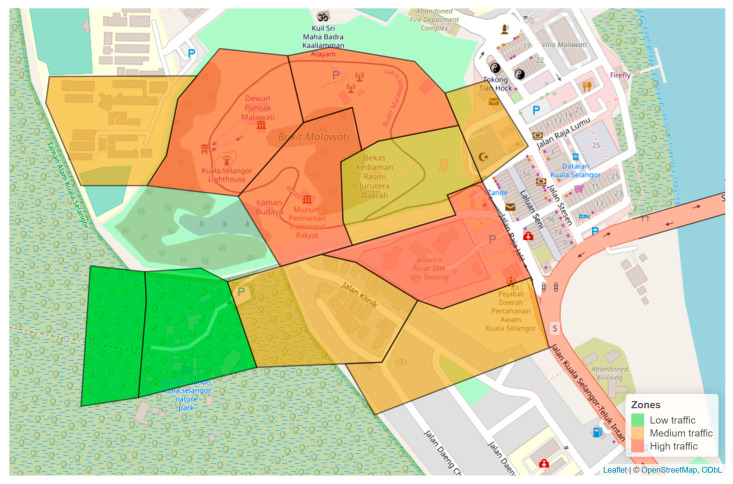
The study area for instantaneous scan sampling was carried out in Kuala Selangor, with zones designated as low, medium, and high traffic.

The site was divided into three zone types: high, medium, and low, loosely defined by the level of human interaction the macaques were likely to experience. Zones with active daily exposure to people (>100 people), either tourists or residents, were identified as high traffic. Zones with lower human activity levels (50–100 people) with people visiting less frequently, so the exposure to monkeys was intermediate, was identified as medium traffic. Zones where human presence was infrequent (<50 people), having a reduced chance of interactions with monkeys, were classified as low traffic, Figure 1. These zones were determined based on initial interviews with residents on the visitor numbers and common activities that occurred in the area. The research team then followed up with observations to finalize the zone distribution. Observations in each zone took place for an equal amount of time. The zones were also divided based on realistic boundaries, e.g., a main road. 

### 2.2. Behavior Sampling Method 

The study species were observed daily from 9:00 to 17:00 from August to November 2015. Instantaneous scan sampling [39] was carried out at time points with varying intervals, usually spanning 3 to 11 min over the time to maintain statistically independent measurements [40]. Data collection was divided to allow observations to be represented in all zones and time periods, which were early morning (9:00–10:29), late morning (10:30–11:59), afternoon (12:00–13:59), early evening (14:00–15:29), and late evening (15:30–17:00). Data were recorded manually using an ethogram consisting of 29 behaviors. A total of 1761 scans were conducted across all time points, with the highest number of individuals observed being 117. The ethogram used for data collection can be found in Supplementary Material Table S1. These observed behaviors were further categorized into ‘Aggression, Self-grooming, Feeding Natural, Feeding Unnatural, Inactive, Affiliative Interaction, Sexual, and Travel’ for analysis. Structures that the LTMs were occupying were recorded and categorized as either ‘Anthropogenic’, human-made structures, e.g., roads, poles, fences, rooftops, or ‘Natural’, naturally occurring structures, e.g., trees and grass grounds. This data were noted collectively instead of individually, meaning if three individuals were on the ground grooming, it was noted as G1, G2, and G3—ground. Only one individual carried out data collection throughout the study. The observer could not be blinded from data collection due to the need to know locations in Kuala Selangor. Minimizing any effect of the observer on LTM behavior was achieved by keeping a minimum distance of 4 m from the monkeys and, prior to data collection, habituating them to the observer’s presence daily for 2 weeks. Habituation occurred through repeated neutral exposure to the observer, resulting in a lack of interest or fear of the observer, and consequently, the observer’s presence was ignored [32].

### 2.3. Data Analysis 

To depict LTM behaviors across different factors such as time of day, zone, presence of humans, and weather conditions, the total number of each behavior was counted, and the percentage was calculated by dividing the frequency by the total number of behaviors observed within said factor considered (e.g., the total number of inactive behavior observed in early morning divided by the total number of behaviors observed in the early morning). Bootstrapping was employed to generate 1000 replicant datasets by resampling with replacement, ensuring each had an equal number of entries as the original dataset. This process enabled the calculation of the mean, along with the upper and lower bounds of the 95% confidence interval. 

A generalized linear model was utilized to explore the relationship between different covariates and the occurrence of select behaviors (See Supplementary Material Table S2). The binomial family of the logit link function was used due to the binary nature of the response variable (occurrence and non-occurrence of a behavior). In the case of zones of traffic, the model uses low-traffic zones as a reference and compares the medium and high-traffic zones to the reference. The estimated odds ratio captures the strength of the association, and a *p*-value threshold of 0.05 was used to assess statistical significance. McFadden’s R-squared was calculated for each model to assess how much variation in the data can be explained by the variables considered. The statistical calculations were conducted using R version 4.0.3 [41], and all data were visualized using the ggplot2 package [42]. Data and R code used for analysis can be accessed at: https://github.com/MabEntez/Human-macaque-interactions (accessed on 27 December 2023). 

## 3. Results 

**Figure 2:**
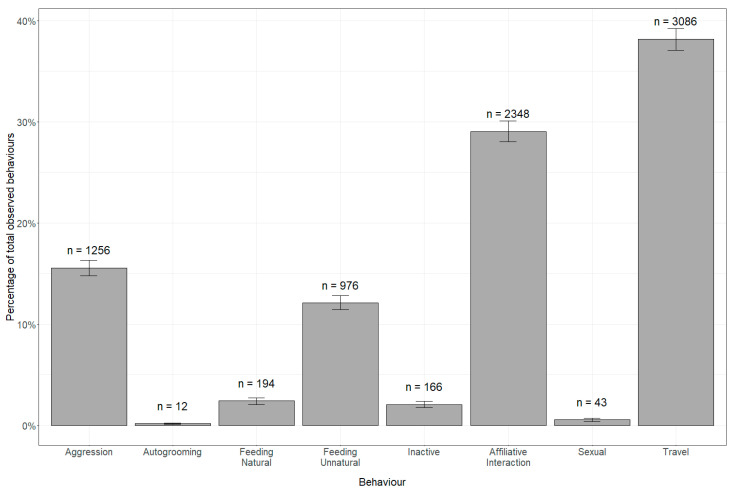
Activity budget of long-tailed macaques (*Macaca fascicularis*) in Kuala Selangor after bootstrapping with n showing the total number of observations for each category and error bars showing the upper and lower 95% quantiles.

In the study area, LTMs were observed to be traveling, inactive, interacting with other monkeys, and feeding naturally in 94.9% of the scans, with self-grooming, unnatural feeding, sexual, and aggression-related activities making up the rest of the observed scans, Figure 2. Of all unnatural feeding, 10 (6%) instances were LTMs being provisioned directly by humans, and out of all inactive LTMs, 1269 (54%) were ‘alert inactive’.

**Figure 3:**
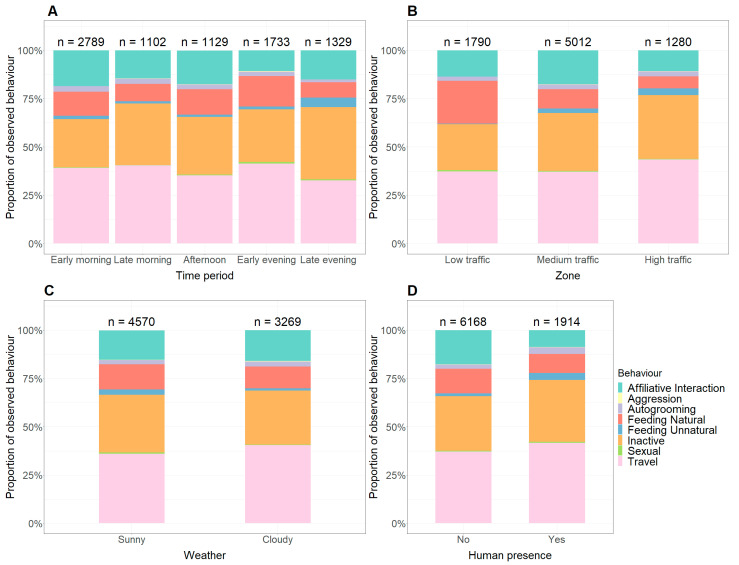
Comparing activity budget proportions of long-tailed macaques in Kuala Selangor (with n showing the total number of observations) in relation to time of day (**A**), zone (**B**), weather (**C**), and presence of humans (**D**).

The majority of LTM observations occurred in the early morning (35.5%) when compared to the late morning (13.6%), afternoon (13.9%), early evening (21.4%), and late evening (16.4%). These numbers are not representative of the number of individuals present but the amount of time LTMs spend in the area of observation. LTMs observed in previous scans that have not traveled out of sight would be counted multiple times. Between each time period, the activity budget of behaviors is relatively similar, with travel (Mean: 37.8%, Range: 32.6–41.3%), inactivity (Mean: 30.2%, Range: 24.9–37.6%), affiliative interactions (Mean: 15.2%, Range: 10.6–18.5%), and natural feeding (Mean: 11.7%, Range: 7.9–15.8%) making up the majority of activity budget, Figure 3A. Aggression (Mean: 0.2% Range: 0.1–4.0%) and unnatural feeding (Mean: 2.1% Range: 1.2–5.0%) are shown to be at their highest in the early evening and late evening, respectively, whereas natural feeding was higher in the early evening and early morning. 

Within the designated zones, most LTMs were observed in medium traffic, Figure 3B. A higher number of LTMs was observed in high and medium traffic zones than in low. Behaviors within each area designation were consistent throughout, with a notable exception being a much higher proportion of natural feeding being observed in medium traffic zones (22.1%) compared to low (10.0%) or high (6.2%) traffic zones. LTMs spent more time feeding (naturally and unnaturally) in low-traffic zones (23.45%) compared to medium (12.31%) and high-traffic zones (9.68%). 

The weather did not alter the activity budget of the LTMs extensively, Figure 3C. LTMs performed more active behaviors when the weather was sunny; for example, feeding naturally occurred at 13.1% when sunny compared to 11.3% when cloudy. 

Human presence was found to affect LTM feeding and affiliative interaction with other monkeys. LTMs fed naturally 9.9% of the time in the presence of humans compared to 12.8% when people were not around, Figure 3D. Furthermore, LTMs interacted with conspecifics less in the presence of humans at 8.6% of the time compared to 17.7% of the time without human presence. 

**Figure 4:**
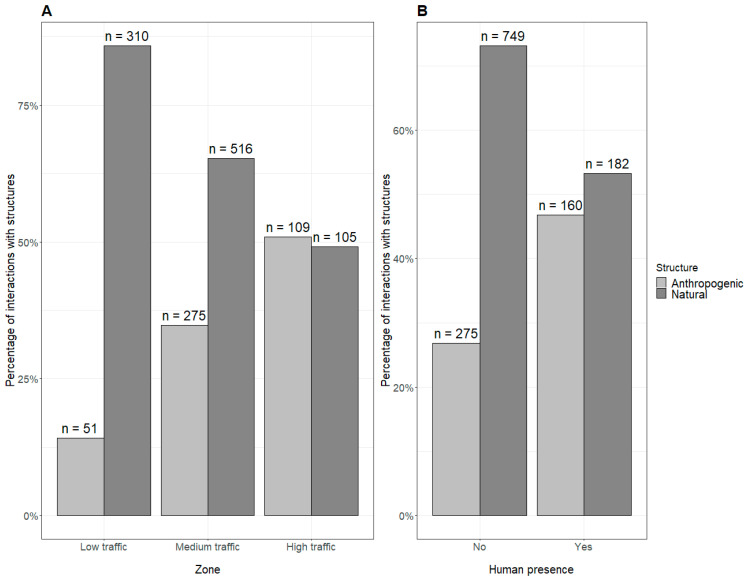
Comparing structure usage of long-tailed macaques in Kuala Selangor (with n showing the total number of observations) in relation to zones (**A**) and presence of humans (**B**).

Observations from across the zones show that the proportion of anthropogenic structure use increases as human traffic increases, Figure 4A. Additionally, LTMs interacted more with anthropogenic structures in the presence of humans (46.8%) compared to when humans were not present (26.9%), Figure 4B. 

The odds ratio, *p*-value, and McFadden’s R-squared values calculated for selected behaviors are shown in Table 1. McFadden’s R-squared in all cases was small, suggesting that the variables considered do not capture the variation in the response variable. For example, much of the frequency of inactive behavior is positively influenced by an odds ratio of 0.56 in a high-traffic area compared to a low-traffic area. This association is statistically significant with a *p*-value of <0.001. McFadden’s R2 for inactive behavior indicates that 0.0359 (3.59%) of the variation in the data can be explained by the variables used in the model. 

## 4. Discussion 

Recognizing the impact of urbanization, tourism, and increased human presence on the daily activity of LTMs is an important step toward understanding human–primate interaction. The overall activity budget of the LTMs showed that the vast majority of the time (94.9%) was spent traveling, being inactive, interacting with other monkeys, and feeding naturally. This falls in line with observations of Hambali et al. [14], which were conducted in the low human traffic zones of the study area described here, and Ruppert et al. on the activity budget of pig-tailed macaques (*Macaca nemestrina*) [43]. LTMs were found to spend more time feeding on unnatural food during times when humans were present in high-traffic zones, consistent with studies done in Malaysia and Singapore [27,44] with a surge of unnatural feeding in the late evening. LTMs spent the highest amount of time foraging for food (natural and anthropogenic) in the low-traffic zones where a reduced number of people and anthropogenic food were accessible. 

Although direct provisioning was observed to be low (6.5%), LTMs fed more on anthropogenic food (found in bins or the environment) in zones of high human traffic (medium and high). Significant positive correlations were found between unnatural feeding, anthropogenic structure usage, and human presence and medium traffic zones. Additionally, natural feeding was negatively correlated with medium and high-traffic zones. Therefore, LTMs might have made some association between humans and anthropogenic food sources, and even without active provisioning, they may seek out unnatural food when people are present. This is consistent with Knight [45], who reported that animals start to expect food in areas where tourists are known to hand out food. Furthermore, studies in Indonesia have found that LTMs that perform robbing and bartering behavior were strongly associated with high exposure to tourists [46]. Similarly, the LTMs in Kuala Selangor who spend more time in tourist zones could potentially develop robbing and bartering behavior, which would further increase the complaints of LTMs. 

Human–animal conflict in Kuala Selangor is recognized from the many complaints that centre around LTM and relate to their endeavors to obtain anthropogenic food (e.g., foraging bins for food) [27]. Despite this, aggressive behaviors were extremely low, which is consistent with observations about LTM in Sepilok Orangutan Rehabilitation Centre, Malaysia [25] and in Gunung Keriang Recreational Park, Kedah, Peninsular Malaysia [47]. This suggests that public perception fuels conflict mitigation strategies, which include lethal control: 97,200 LTM were culled in 2012 [48]. Culling as a control measure does little to dispel conflict as habituation and habitat loss still drive anthropogenic food sourcing. However, it potentially reduces the number of LTMs living in urban habitats. A consideration to reduce human–primate conflict and thus reduce the number of complaints would be to stop the LTMs from associating humans with anthropogenic food. Alternative interventions addressing this could be in the form of stopping the unnatural feeding of monkeys by cleaning up any leftover food (before late evening when anthropogenic feeding is highest) and making sure monkeys do not have access to food by using bin latches or monkey-proof bins. 

LTM are an edge species and are able to exploit the fragmented environments produced by anthropogenic land use [49]. Despite being broadly perceived as successfully adapting to urban environments, it is important to consider how urban living primates are affected by the presence of humans. The difference between zones in this study provides a strong plausible link that human presence influences LTM feeding behavior. As animals can only engage in one activity at a given time, the time they can spend on different activities is restricted throughout the day. This time constraint leads to trade-offs; as LTMs change behaviors to adapt to their setting, this will inevitably eat away at the time when they could be doing other beneficial activities [24]. A similar study found that field mice (*Apodemus sylvaticus*) showed a higher tolerance and less aggressive behavior in urban settings, supporting the ‘trade-off’ [50]. 

The behavior observed in this study, 54%, was ‘alert inactivity’ where LTMs are inactive and monitoring their surroundings. Vigilance is considered costly as it is not carried out alongside other activities such as foraging, limiting the amount of time available for said activity, and raising questions about the animal’s welfare. Vigilant behavior is carried out to monitor the environment for predators [51], and hence, the frequency of vigilance varies with those factors that influence predation risk. Tourists may be perceived as predators, similar to those present in the wild, promoting instinctive anti-predator behaviors [52]. Md-Zain et al. [53] report ‘staring’ as being the third most frequent interaction between silvered-leaf monkeys and tourists in Kuala Selangor. LTMs spent an increased amount of time inactive in higher human traffic zones and when in the presence of humans. Inactivity was found to have a positive association in medium and high traffic zones as well as human presence, an indicator that LTMs are spending more time conducting vigilant behaviors not only in the presence of humans but in areas where humans are more likely to be present [51]. Overall, little variation was detected in behavior concerning weather changes. As only sunny and cloudy weather was observed (observations were stopped in the event of rain), this lack of significant change to the LTM activity budget may be explained by the absence of more disruptive weather (i.e., rain or strong winds) within the data. Anthropogenic structure usage was found to be negatively correlated with inactive behavior. In this case, LTMs are spending time being wary and vigilant around humans and are spending less time conducting more positive behaviors for their well-being, such as socializing [24]. 

Affiliative interaction includes behaviors such as social play, which are defined as more than two individuals interacting [54] in a way that influences the others’ behavior [55]. This is affected by habitat type, resource availability, and social dynamics [56,57]. Affiliative interaction is critical to the group well-being of primates [58,59]. Affiliative interaction establishes and maintains long-term bonds [59,60,61,62], group cohesion, and social stability [63,64]. When the environment is compatible with a good quality of life for that species, the frequency of play is higher than when the habitat is inadequate [55]. Therefore, suitable environmental conditions can be assessed by monitoring the prevalence of play behavior [65]. Anthropogenic structure and human presence were shown to have a negative association with affiliative interaction. In contrast, interaction was positively associated with medium and high-traffic zones. Although this information might sound contradictory, this could be due to the time constraints previously mentioned. Importantly, human presence is associated with a reduction in affiliative interaction and an increase in inactivity, meaning the time they would be spending interacting is now spent inactive. This can be explained by the idea that the LTMs do not spend as much time foraging due to the more readily available anthropogenic food sources, making more time available for other activities. Overall, this shows that LTMs have deviated from their natural behavior patterns due to changes in their environment, emphasizing the effect of human presence on the reduction in LTM social interaction. 

Similar to trends in natural feeding, anthropogenic structure use was found to be increased in zones of higher human traffic and human presence. It could be speculated that this is due to LTMs associating anthropogenic structures with anthropogenic food, or this could be some form of coping mechanism in response to increased human presence and adapting to an urban environment. More work needs to be done to study the LTM coping mechanisms in this tourism space where trade-offs occur [35]. 

## 5. Conclusions 

Long-tailed macaques are known to be adaptive and opportunistic, as they alter their behaviors to achieve resources to survive. This study shows changes in the natural behavior of long-tailed macaques in areas with high human activity. There was more inactivity, decreased affiliative interaction, and consumption of anthropogenic food when humans were present and in high-activity areas, even when provisioning was not happening. These altered behaviors of the long-tailed macaques in high human traffic zones often drive complaints of pest behavior projected from a lack of tolerance by people towards them. Moreover, an increase in altered behaviors raises questions about animal welfare as it differs from natural activity. Understanding ecological behavior and causes for altered behavior is crucial so that a conservation plan can be developed to reduce human–monkey conflict and generate a long-term mitigation strategy. 

## Figures and Tables

**Table 1:** Multiple logistic regressions using a binomial function identify which factors affect the display of different behaviors by long-tailed macaques. The odds ratio shows the strength of the negative or positive correlation between the variable and the observed behavior. *p*-value denotes significance, with NS showing no significance.

Behavior	Variable	Odds Ratio	*p*-Value	McFadden’s R2
Travel	Intercept	0.00	<0.001	0.0398
	Anthropogenic structure	1.39	<0.001	
	Sunny weather	0.85	<0.001	
	Human presence	1.06	NS	
	Medium traffic area	0.94	NS	
	High traffic area	1.07	NS	
Inactive	Intercept	0.30	<0.001	0.0359
	Anthropogenic structure	0.82	<0.001	
	Sunny weather	1.09	<0.05	
	Human presence	1.12	<0.05	
	Medium traffic area	1.49	<0.001	
	High traffic area	0.56	<0.001	
Affiliative Interaction	Intercept	0.21	<0.001	0.057
	Anthropogenic structure	0.57	<0.001	
	Sunny weather	0.90	<0.05	
	Human presence	0.49	<0.001	
	Medium traffic area	1.67	<0.001	
	High traffic area	1.54	<0.001	
Feeding Natural	Intercept	0.26	<0.001	0.0558
	Anthropogenic structure	1.16	<0.05	
	Sunny weather	1.16	<0.01	
	Human presence	1.17	<0.05	
	Medium traffic area	0.34	<0.001	
	High traffic area	0.18	<0.001	
Feeding Unnatural	Intercept	0.00	<0.001	0.1063
	Anthropogenic structure	12.18	<0.001	
	Sunny weather	2.61	<0.001	
	Human presence	1.25	<0.001	
	Medium traffic area	3.39	<0.001	
	High traffic area	3.03	NS	
Self grooming	Intercept	0.03	<0.001	0.0513
	Anthropogenic structure	0.80	NS	
	Sunny weather	0.84	NS	
	Human presence	1.93	<0.001	
	Medium traffic area	0.98	NS	
	High traffic area	0.92	NS	

## Data Availability

The data presented in this study are available on request from the corresponding author. The data are not publicly available due to privacy reasons.

## Supplementary Materials

The following supporting information can be downloaded at: https://www.mdpi.com/article/10.3390/ani14081173/s1, Table S1: Ethogram of long-tailed macaque (*Macaca fascicularis*) behaviour and structure use. Table S2: Multiple logistic regression model response variable and covariates.
