# Supplementary material for: Effect of Human Activity and Presence on the Behavior of Long-Tailed Macaques (Macaca fascicularis) in an Urban Tourism Site in Kuala Selangor, Malaysia
Source: Animals (Basel). 2024 Apr 13;14(8):1173. doi: 10.3390/ani14081173 (PMC11047574; doi:10.3390/ani14081173)
Supplement: Supplementary file 1 [file animals-14-01173-s001.zip › animals-2835560-supplementary.pdf]

Supplementary Table S1: Ethogram of long-tailed macaque (*Macaca fascicularis*) behaviour and structure use.

Behaviour States	Code	Description
**Inactive**		
Inactive resting	IR	Maintaining a posture with no other behaviour being performed. Eyes are shut.
Inactive alert	IA	Maintaining a posture with no other behaviour being performed. Eyes are opened while monitoring around.
Inactive back	IB	Maintaining a posture with no other behaviour being performed with the back turned away. Unable to see monkeys face.
**Travel**	T	Terrestrial or arboreal movement from one place to another done by one or more members in a group.
**Feeding natural**	FO	Searching, manipulating, and consuming natural occurring items/non anthropogenic
**Feeding unnatural**	FON	Searching, manipulating, and consuming items from an unnatural source e.g. trash bins.
**Affiliative Interaction**		
Grooming	G2	One individual combs through another individuals coat using its fingers while picking, scratching or removing objects from the hair. May or may not consume.
Play	P	Wrestling, thumbling, rough playing or chasing involving two or more individuals in a non-aggressive manner.
Object play	OP	Using an object individually or with play mates for purpose of play. Licking or putting to mouth can be seen occasionally but not used predominantly to eat.
Being groomed	BG	An animal is in an inactive position while receiving grooming from another individual or more than one individual.

**Aggression**	X	Aggressive behaviour such as threats, grabbing, fighting, biting etc shown to an individual, a few individuals, humans or other species
**Sexual**	S	Mounting with intromission. The male mounts the female and uses his legs to grip the female and hands are grasping the female's waist. May be accompanied by vocalization.
**Self- grooming**	G	An individual combs it own fur using its fingers while picking, scratching, or removing objects from the hair. May or may not consume.
Solitary play	P1	An individual walks with a bouncy gait, jumps and rolls about sometimes trying to catch its tail. Can be using objects from the environment as well.

Structure	Symbol
**Anthropogenic structure**	
Electric post	e
Picnic table/chair	pt/pc
Roof	r
Roadside divider	rd
Structure	s
Vehicle	v
Building	b
Dustbin	d
Fence	f
Tower	tw
Road	o
Poles	p
**Natural structures**	
Tree	t
Ground	g

Supplementary Table S2: Multiple logistic regression model response variable and covariates.

Response Variable (Behaviour)	Covariate	Reference
Travel	Anthropogenic substrate	Natural substrate only
	Sunny weather	Cloudy weather
	Human presence	Humans absent
	Medium traffic area	Low traffic area
	High traffic area	Low traffic area
Inactive	Anthropogenic substrate	Natural substrate only
	Sunny weather	Cloudy weather
	Human presence	Humans absent
	Medium traffic area	Low traffic area
	High traffic area	Low traffic area
Interaction	Anthropogenic substrate	Natural substrate only
	Sunny weather	Cloudy weather

	Human presence	Humans absent
	Medium traffic area	Low traffic area
	High traffic area	Low traffic area
Feeding Natural	Anthropogenic substrate	Natural substrate only
	Sunny weather	Cloudy weather
	Human presence	Humans absent
	Medium traffic area	Low traffic area
	High traffic area	Low traffic area
Feeding Unnatural	Anthropogenic substrate	Natural substrate only
	Sunny weather	Cloudy weather
	Human presence	Humans absent
	Medium traffic area	Low traffic area
	High traffic area	Low traffic area
Autogrooming	Anthropogenic substrate	Natural substrate only
	Sunny weather	Cloudy weather
	Human presence	Humans absent

	Medium traffic area	Low traffic area
	High traffic area	Low traffic area
